# Genome-Wide Identification and Functional Characterization of *SKP1-like* Gene Family Reveal Its Involvement in Response to Stress in Cotton

**DOI:** 10.3390/ijms26010418

**Published:** 2025-01-06

**Authors:** Zhao Geng, Jianguang Liu, Guiyuan Zhao, Xiangli Geng, Xu Liu, Xingyu Liu, Hanshuang Zhang, Yongqiang Wang

**Affiliations:** 1 Institute of Cotton, Hebei Academy of Agriculture and Forestry Sciences/Key Laboratory of Cotton Biology and Genetic Breeding in Huanghuaihai Semiarid Area, Ministry of Agriculture and Rural Affairs, Shijiazhuang 050000, China; gengzhao1006@163.com (Z.G.); liujianguangbj@126.com (J.L.); zhaogy0302@163.com (G.Z.); adorable0267@126.com (X.L.); 2 Institute of Grain and Oil Crops, Hebei Academy of Agriculture and Forestry Sciences, Shijiazhuang 050000, China; xiangligeng001@163.com; 3 College of Food Science and Biology, Hebei University of Science and Technology, Shijiazhuang 051432, China; liuxingyu2411@163.com

**Keywords:** *SKP1-like*, synteny and collinearity analyses, expression patterns

## Abstract

SKP1 constitutes the Skp1-Cullin-F-box ubiquitin E3 ligase (SCF), which plays a role in plant growth and development and biotic and abiotic stress in ubiquitination. However, the response of the *SKP1-like* gene family to abiotic and biotic stresses in cotton has not been well characterized. In this study, a total of 72 *SKP1-like* genes with the conserved domain of SKP1 were identified in four Gossypium species. Synteny and collinearity analyses revealed that segmental duplication played a major role in the expansion of the cotton *SKP1-like* gene family. All SKP1-like proteins were classified into three different subfamilies via phylogenetic analysis. Furthermore, we focused on a comprehensive analysis of *SKP1-like* genes in *G. hirsutum*. The cis-acting elements in the promoter site of the *GhSKP1-like* genes predict their involvement in multiple hormonal and defense stress responses. The expression patterns results indicated that 16 *GhSKP1-like* genes were expressed in response to biotic or abiotic stresses. To further validate the role of the *GhSKP1-like* genes in salt stress, four *GhSKP1-like* genes were randomly selected for gene silencing via VIGS. The results showed that the silencing of *GhSKP1-like_7A* resulted in the inhibition of plant growth under salt stress, suggesting that *GhSKP1-like_7A* was involved in the response to salt stress. In addition, yeast two-hybrid results revealed that *GhSKP1-like* proteins have different abilities to interact with F-box proteins. These results provide valuable information for elucidating the evolutionary relationships of the *SKP1-like* gene family and aiding further studies on the function of *SKP1-like* genes in cotton.

## 1. Introduction

Plants are recognized for their ability to regulate cellular stability by undergoing protein synthesis and breakdown to adapt to a variety of environmental and biological pressures (such as high temperatures, a lack of water, and diseases) and carry out essential survival functions [1]. The ubiquitin–proteasome system (UPS) and autophagy pathways are two key mechanisms for eukaryotic degradation and protein signal transduction [2,3]. In plants, ubiquitin-mediated proteolysis by the 26S proteasome occurs in response to different environmental stresses and developmental signals. Proteasome-mediated ubiquitination of degradation substrates by the 26S proteasome is catalyzed by three types of enzymes: E1 ubiquitin-activating enzymes, E2 ubiquitin-conjugating enzymes, and E3 ubiquitin ligases. SCF (Skp1-Cullin-F-box) ubiquitin complexes constitute the largest class of E3 enzymes. SKP1-like protein (S-phase kinase-related protein 1), the core part of the SCF complex, can bind to the Cullin (Cdc53) protein and various F-box protein subunits that specify substrates [4].

Previous studies have suggested that *SKP1-like* genes are involved in regulating many basic metabolic reactions of cells, redox reactions, photosynthesis, and reactive oxygen species (ROS) homeostasis and play key roles in plant development and stress response. In *A. thaliana*, the gene *ASK1*, a homolog of *SKP1* (*Arabidopsis SKP1-LIKE 1*), plays a crucial role in controlling plant hormone responses to auxin and jasmonate through the SCF^TIR1^ and SCF^COI1^ complexes [5,6]. Furthermore, studies have shown that *ASK1* is vital for the development of vegetative and floral structures and is necessary for male meiosis. The *Arabidopsis ASK1* homologous gene *LeSKP1* regulates the heat resistance of Lentinus edodes by participating in the auxin signaling pathway, providing a candidate gene for cultivating heat-tolerant Lentinus edodes [7]. In *Glycine max*, *GmSKP1* increased resistance to *Phytophthora sojae* by activating defense-related PR genes [8]. Hu et al. [9] suggested that the overexpression of the *GhSKP1* gene in tobacco delayed seed germination and shortened the main root length by increasing ABA sensitivity, indicating that the *GhSKP1* gene serves an important function in plant growth and development and that the *SKP1* gene may be functionally conserved. *GhACIF1* (F-box protein) interacts with *GhSKP1*, and it is evident that *GhSKP1* can be a component of the SCF complex and plays a role in multiple physiological functions [10].

Cotton (*Gossypium* spp.) is a major cash crop worldwide that is often subjected to biotic and abiotic stresses such as salt, cold stress, drought stress, and *Verticillium wilt* infection during its growth process. The primary types of cultivated cotton in my nation include *G. arboreum*, *G. hirsutum*, and *G. barbadense*. *G. arboreum* is classified as diploid, whereas *G. hirsutum* and *G. barbadense* are allotetraploid. Furthermore, a significant diploid variety, *G. raimondii*, exists within the cotton species. Despite its minimal production of cotton fibers, it holds considerable importance in the evolutionary development of cotton species. To further characterize the stress tolerance of the *SKP1-like* gene family in cotton, we carried out a comprehensive analysis of this gene family, including the structure, phylogeny, duplication events, and expression patterns of the *SKP1-like* gene family in four *Gossypium* species. In addition, we analyzed the homoeology, chromosomal distributions, and collinear relationships among *G. hirsutum* (Gh), *G. barbadense* (Gb), *G. arboreum* (Ga), and *G. raimondii* (Gr). Finally, we investigated the structure, promoter, and expression patterns of the *SKP1-like* gene family in various tissues and in response to biotic-abiotic stresses via the use of public data. These results may provide a basis for a comprehensive understanding of the functions of *SKP1-like* genes in biotic and abiotic stress responses in cotton.

## 2. Results

### 2.1. Identification and Distribution of SKP1-like Family Members in Four Gossypium Species

A total of 72 *SKP1-like* genes were initially identified by Hmmer, including 22, 16, 22, and 12 genes in *G. hirsutum* (*GhSKP1-like*), *G. raimondii* (*GrSKP1-like*), *G. barbadense* (*GbSKP1-like*), and *G. arboreum* (*GaSKP1-like*), respectively (Additional file 1: Table S1). The sequences of *SKP1-like* family members vary greatly, with ORF sequences ranging from 327 bp to 1083 bp and molecular weights ranging from 15.5 kDa to 61.7 kDa. ExPASy analysis revealed that the theoretical isoelectric point distribution of SKP1-like proteins is between 4.09 and 7.74, indicating that there is little difference in the physicochemical properties of members of the *SKP1-like* family. In addition, subcellular localization prediction of the protein indicated that all SKP1-like proteins are present in the nucleus, and we also found that these proteins are hydrophilic (Additional file 1: Table S2).

**Figure 1:**
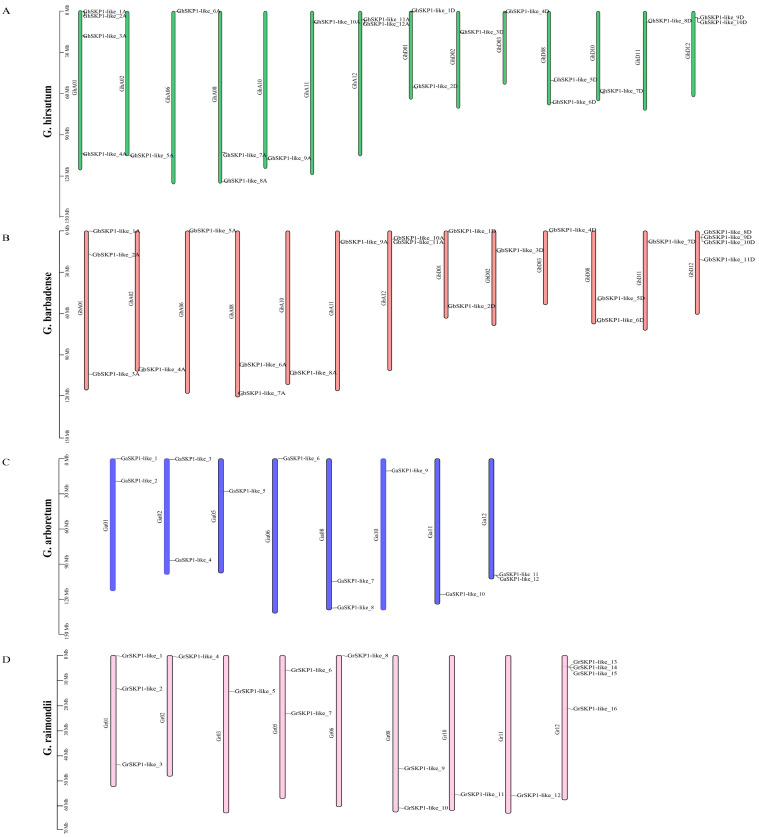
Chromosomal positions of *SKP1-like* genes from four cotton species with gene IDs shown on the right side. The vertical bar on the left side represents the position of the gene and the length of the chromosome. (**A**): *G. hirsutum*. (**B**): *G. barbadense*. (**C**): *G. arboretum*. (**D**): *G. raimondii*.

To better understand the chromosomal distribution of the *SKP1-like* genes, we created physical maps of the chromosomal distribution of the *SKP1-like* gene family in the four *Gossypium* species. A total of 72 *SKP1-like* genes were distributed on 44 chromosomes in four *Gossypium* species (Figure 1, Additional file 1: Table S3). In *G. hirsutum*, 12 and 10 genes were located on the A subgenome and D subgenome, respectively. Among these genes, chromosome A contains the most genes, with four *SKP1-like* genes. In *G. barbadense*, 22 genes were distributed on thirteen chromosomes, of which 11 *SKP1-like* genes were on the A subgenome and another 11 *SKP1-like* genes were on the D subgenome. In *G. arboreum*, 12 genes were distributed on eight chromosomes. In *G. raimondii*, 16 genes were distributed on nine chromosomes.

The number of *SKP1-like* genes in the tetraploid is less than the sum of the number of *SKP1-like* genes in the two diploids, suggesting that certain *SKP1-like* genes were lost during the evolutionary transition from diploid to tetraploid. However, there remains a strong correspondence between subgenomes A and D in the tetraploid and the A and D genomes in the diploids.

### 2.2. Phylogenetic Analysis of SKP1-like Genes

**Figure 2:**
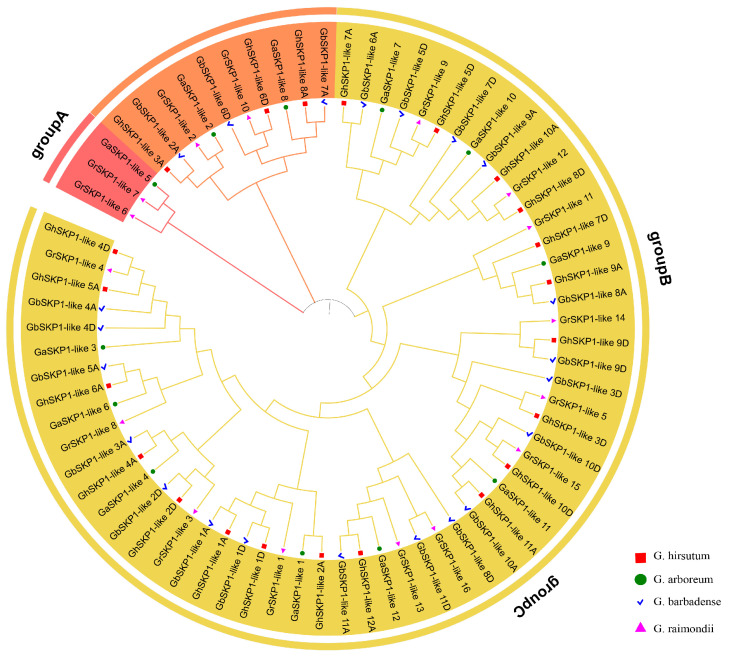
The phylogenetic tree constructed using the neighbor-joining method, based on analysis of SKP1-like protein sequences.

To analyze the evolutionary relationships of *SKP1-like* genes in *G. hirsutum*, *G. arboreum*, *G. barbadense*, and *G. raimondii*, a rooted neighbor-joining phylogenetic tree was constructed using full-length amino acid sequences. All the *SKP1-like* family members can be divided into three groups, Groups A to C (Figure 2). Group A has the fewest number of members, with only two *GrSKP1-like* members and one *GaSKP1-like* member. Group B contains 10 members. Group C is the largest branch, containing 59 members. However, Group C has many internal branches. We noted that the SKP1-like proteins in the A subgenomes of *G. hirsutum* and *G. barbadense* clustered with those of G. arboreum, whereas the SKP1-like proteins in the D genomes of *G. hirsutum* and *G. barbadense* clustered with those of *Graimondii*. These results confirmed the conclusion that *G. hirsutum* and *G. barbadense* are the result of crosses between *G. arboreum* and *G. raimondii* and suggested that *SKP1-like* genes were relatively conserved during evolution. In addition, group A contained only genes from *G. arboreum* and *G. raimondii*, suggesting that genes in group A were lost during the formation of tetraploids.

### 2.3. Collinearity Analysis of SKP1-like Genes in Four Gossypium Species

**Figure 3:**
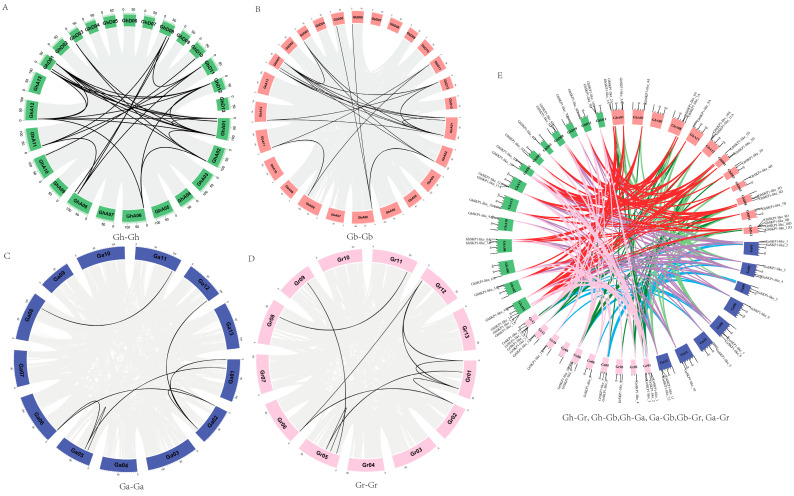
The relationship of gene duplication among *SKP1-like* genes across four cotton species is depicted through distinct inter-genomic and intra-genomic syntenic regions, each represented in different colors. The duplicated gene pairs syntenic relationships were established using the following combinations. (**A**) (Gh-Gh), (**B**) (Gb-Ghb), (**C**) (Ga-Ga), (**D**) (Gr-Gr), and (**E**) (all, Ga-Gr, Ga-Gh, Ga-Gb, Gr-Gh, Gr-Gb, and Gh-Gb).

To reveal the homologous relationships of the *SKP1-like* gene family members in the four cotton species, gene duplication events were analyzed via MCScanX. In this study, by comparing the genomes and subgenomes of Gh-Gh, Gb-Gb, Gr-Gr, Ga-Ga, Ga-Gr, Ga-Gh, Ga-Gb, Gr-Gh, Gr-Gb, and Gh-Gb, we identified a total of 373 pairs of linear or paralogous genes (Figure 3A–D). There were 25, 49, 44, 54, 39, and 82 linear gene pairs that replicated in Ga-Gr, Ga-Gh, Ga-Gb, Gr-Gh, Gr-Gb, and Gh-Gb, respectively (Figure 3E, Additional file 1: Table S4). In addition, 3, 2, 1, and 1 tandem duplication events were observed in *G. hirsutum*, *G. barbadense*, *G. arboreum*, and *G. raimondii*, respectively. The results indicated that the *SKP1-like* genes of tetraploid cotton (AD-genome) mainly originated from interspecific crosses between the diploid cotton *G. arboreum* (A-genome) and *G. raimondii* (D-genome) (Figure 3, Additional file 1: Table S5). The major causes of gene amplification in *G. arboreum* and *G. raimondii* were whole-genome duplication events or fragment duplication events.

### 2.4. Gene Structure and Motif Analysis of SKP1-like in G. hirsutum

**Figure 4:**
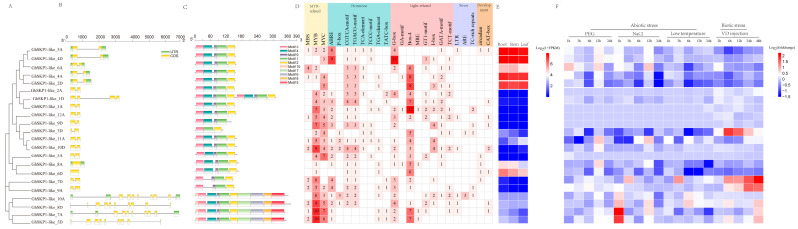
Analysis of gene structure, conserved protein motifs, and promoters in *GhSKP1-like* genes. (**A**) The phylogenetic tree was created utilizing the NJ method, which relied on the full-length sequences of GhSKP1-like proteins. (**B**) Ten types of *GhSKP1-like* conserved motifs, the sequence information for each motif is provided in Table S5. (**C**) Exon–intron structure of *GhSKP1-like* genes. Untranslated regions, exons, and introns are shown as light green boxes, light yellow boxes, and horizontal lines, respectively. (**D**) Cis-acting elements were identified in the 2000 bp promoter regions of *GhSKP1-like* genes, and these regulatory elements were classified into 24 distinct types, each represented by different colors. The lower axis indicates the length of the gene. (**E**,**F**) Heatmap depicting the expression patterns of *GhSKP1-like* genes in different tissues and responses to abiotic and biotic stresses.

*G. hirsutum* is the most important cotton species, accounting for approximately 90% of cotton commerce worldwide. Therefore, we focused on a comprehensive analysis of *SKP1-like* genes in *G. hirsutum*. Gene structure plays a vital role in the evolution of multiple gene families. Neighbor-joining phylogenetic tree and gene structure analyses revealed that *GhSKP1-like* genes with similar genetic structures clustered together. The number of introns varied from 0 to 9. *GhSKP1-like_8A* and *GhSKP1-like_6D* are intronless, *GhSKP1-like_1D* has 2 introns, *GhSKP1-like_5D* contains 8 introns, and 15 (68.19%) genes contain 1 intron, and *GhSKP1-like_10A*, *GhSKP1-like_8D*, and *GhSKP1-like_7A* contain the most introns, with up to 9 (Figure 4C).

The MEME program was used to identify the conserved motifs in the complete amino acid sequences of the GhSKP1-like proteins. Moreover, the predicted motifs were annotated. A total of ten conserved motifs, varying in length from 8 to 50 amino acids, were identified (Figure 4B, Additional file 1: Table S6). Each GhSKP1-like protein contained different numbers of conserved motifs, ranging from 3 to 10. *GhSKP1-like_3D* has only three conserved motifs, whereas *GhSKP1-like_10A*, *GhSKP1-like_8D*, *GhSKP1-like_7A*, and *GhSKP1-like_5D* each contain ten conserved motifs. Motif analysis of GhSKP1-like proteins revealed that, in general, closely related SKP1-like proteins from the same phylogenetic clade presented similar motif distributions and sequences, implying functional similarities.

### 2.5. GhSKP1-like Gene Promoter and Expression Pattern Analysis

To better understand the regulatory mechanisms of *GhSKP1-like* genes, we identified the cis-acting elements of each gene via the online tool PlantCARE. A total of 24 types of cis-regulatory elements were identified. The promoters primarily contain cis-acting elements associated with hormone regulation, MYB-related elements, developmental processes, and responses to stress and light (Figure 4D, Additional file 1: Table S7). The promoters of all 22 *GhSKP1-like* genes contained hormone-responsive, light-related, and MYB-related elements. In addition, we identified 10 gene promoters containing stress-responsive elements and 10 gene promoters containing development-related elements.

To explore the expression patterns of the *GhSKP1-like* genes, we analyzed the expression of the *GhSKP1-like* genes in the roots, stems, and leaves as well as the expression of the *GhSKP1-like* genes in response to abiotic (low temperature, PEG, NaCl) and biotic (injection of Verticillium dahlia) stress treatments on the basis of transcriptome data from the GRAND database (https://grand.cricaas.com.cn/ (accessed on 31 December 2019)) and the GSA database [11] (Additional file 1: Table S8). Notably, genes from the same branch with similar gene structures mostly presented similar expression patterns. Five genes, namely, *GhSKP1-like_5A*, *GhSKP1-like_4D*, *GhSKP1-like_6A*, *GhSKP1-like_4A*, and *GhSKP1-like_2A*, are highly expressed in the roots, stems, and leaves, and expression tends to decrease in response to cold and *Verticillium dahliae* (VD) stresses. *GhSKP1-like_7D* and *GhSKP1-like_9A* were not expressed in the three cotton tissues. However, these two genes were strongly induced after VD injection. The four genes with the most motifs, *GhSKP1-like_10A*, *GhSKP1-like_8D*, *GhSKP1-like_7A*, and *GhSKP1-like_5D*, were significantly upregulated at 1 h and then downregulated within 3–24 h after salt stress. In addition, it appears that there is no relationship between the cis-regulatory elements and the expression bias observed in *GhSKP1-like* genes (Figure 4E,F). This result has also been reported in other studies [12,13,14], indicating that a complex regulatory mechanism controls the expression of genes.

On the basis of transcriptome data analysis, four genes (*GhSKP1-like_4A*, *GhSKP1-like_7A*, *GhSKP1-like_9A*, and *GhSKP1-like_3D*) were selected for qPCR analysis under 200 mM salt treatment and VD injection. The results of the salt treatment revealed that the expression level of *GhSKP1-like_7A* increased 2-fold after 6 h of salt treatment, whereas the expression levels of both *GhSKP1-like_4A* and *GhSKP1-like_9A* did not obviously change. However, the expression of the *GhSKP1-like_3D* gene was significantly reduced. After VD inoculation, the expression levels of *GhSKP1-like_4A* and *GhSKP1-like_7A* decreased gradually at 12 h but then stabilized at 24 h. *GhSKP1-like_9A* expression gradually increased, with a 3-fold increase at the highest level at 48 h. The expression of *GhSKP1-like_3D* also increased at 2 h, peaked at 6 h, and then decreased at 12 h but was still greater than the normal level at 48 h (Figure 1, Additional file 1: Table S9). Overall, the qPCR results are consistent with the transcriptome analysis results. These results indicate that the *GhSKP1-like* genes play important roles in the response to biotic and abiotic stresses.

### 2.6. Verification of the Function of the GhSKP1-like Gene in Response to Salt Stress

**Figure 5:**
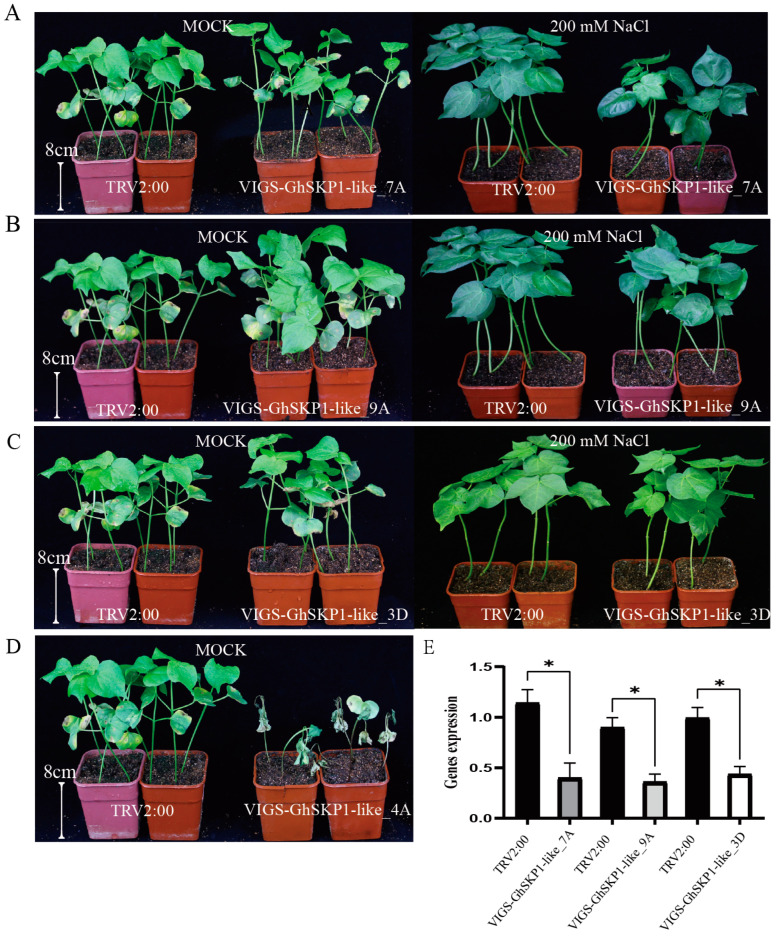
Salt tolerance analysis of the *GhSKP1-like_4A*, *GhSKP1-like_7A*, *GhSKP1-like_9A*, and *GhSKP1-like_3D* silencing cotton plants. (**A**–**D**) Representative phenotypes of TRV:00 (CK) and silenced GhSKP1s plants after 2 weeks of salt treatment, (**E**) the expression of *GhSKP1-like_7A*, *GhSKP1-like_9A*, and *GhSKP1-like_3D* determined by qRT–PCR after the VIGS injection. “*” indicates that the gene expression level was significantly lower in silenced plants (*p* < 0.05) than in TRV2:00 plants.

To explore the functions of the *GhSKP1-like* genes in response to salt stress, four genes (*GhSKP1-like_4A*, *GhSKP1-like_7A*, *GhSKP1-like_9A*, and *GhSKP1-like_3D*) were silenced via VIGS. The silencing of target genes in plants was verified via qRT–PCR (Figure 5E). The results revealed that silencing of the *GhSKP1-like_4A* gene led to death (Figure 5D). Notably, the expression of the *GhSKP1-like_4A* gene is high in various tissues, implying that the *GhSKP1-like_4A* gene is necessary for cotton growth and is a housekeeping gene. When the expression of the *GhSKP1-like_7A* gene was reduced, the plants were significantly inhibited compared with the control plants after salt stress, with lower plant height and biomass (Figure 5A), whereas the other two genes (*GhSKP1-like_9A* and *GhSKP1-like_3D*) were not significantly different from the control (Figure 5B,C). These results indicate that the *GhSKP1-like_7A* gene may play an important role in the response to salt stress, and its function merits further study.

### 2.7. Y2H Validation of the Interaction Between the Cotton SKP1-like Protein and F-Box Protein

**Figure 6:**
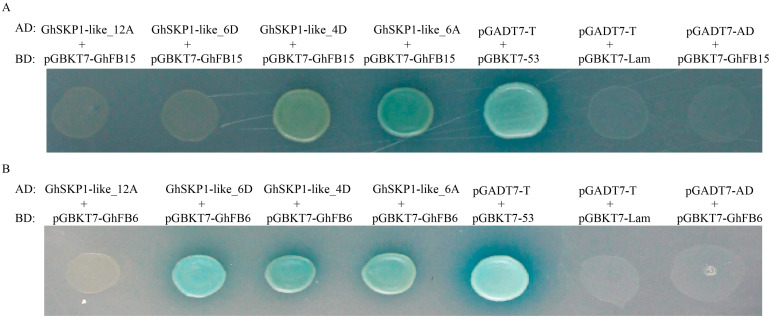
Interaction assay for GhSKP1-like and F-box proteins. (**A**) GhFB15. (**B**) GhFB6.

A prior study showed that the F-box protein from wheat could interact with the protein similar to SKP1-like [15]. To clarify the interaction between GhFBX and GhSKP1-like proteins, we used yeast two-hybrid technology for analysis. On the basis of the expression of GhSKP1-like genes in tissue, we selected 4 *GhSKP1-like* genes (*GhSKP1-like_4D*, *GhSKP1-like_6A*, *GhSKP1-like_6D*, and *GhSKP1-like_12A*) and two F-box genes (*GhFB15* and *GhFB6*) for hybridization analysis. The *GhFB15* gene has been reported to be related to salt tolerance [16], and the *GhFB6* gene may be related to disease resistance according to our research. The results revealed that the AD-*GhSKP1-like_4D*/BD-*GhFB15*, AD-*GhSKP1-like_6A*/BD-*GhFB15*, AD-*GhSKP1-like_6D*/BD-*GhFB6*, AD-*GhSKP1-like_4D*/BD-*GhFB6*, and AD-*GhSKP1-like_6A*/BD-GhFB6 combined strains grew well on selective media with blue colonies, indicating that they can interact. *GhSKP1-like_12A* does not interact with these two F-box genes (Figure 6). The results indicate that the F-box gene can interact with different SKP genes and that the SKP1-like gene can also interact with different F-box genes. The functions of the SCF complex formed by SKP and the F-box are diverse.

## 3. Discussion

### 3.1. Evolution of SKP1-like Genes in Cotton

*SKP1-like* genes encode an E3 ubiquitin ligase member of the SCF complex. Among them, the specific recognition of target proteins by E3 plays a decisive role in the ubiquitin-regulated pathway [17]. In this study, we identified and analyzed the *SKP1-like* genes of four species, namely, *G. hirsutum*, *G. barbadense*, *G. arboreum*, and *G. raimondii.* Two allotetraploid cotton varieties, *G. hirsutum*, and *G. barbadense*, are hypothesized to have originated from a common ancestor through an interspecific hybridization event between *G. raimondii* and *G. arboretum* [18,19]. In this study, a total of 72 *SKP1-like* genes were obtained. In addition, a total of 373 segmentally duplicated gene pairs were identified in the four cotton species; however, only 7 tandemly duplicated gene pairs were identified, indicating that the expansion of the SKP1-like gene family in the cotton genome has resulted from whole-genome duplication. Chromosomal distribution and collinearity analyses revealed that *SKP1-like* family members had good collinearity among the four cotton species. Theoretically, the total number of *SKP1-like* genes in these two allotetraploid cottons should be equal to the sum of the number of *SKP1-like* genes in *G. raimondii* and *G. arboreum*. However, the number of *SKP1-like* genes identified in *G. hirsutum* and *G. barbadense* was lower than that in *G. arboreum* and *G. raimondii* combined. This suggested that some genes were lost or pseudogenized during the process of polyploidization, such as the *GrSKP1-like_6* and *GrSKP1-like_7* genes on Chr5 of *G. raimondii* and the *GaSKP1-like_5* gene on Chr5 of *G. arboreum*, which were lost in tetraploids. In addition, some tandem duplication events and gene loss events of *SKP1-like* genes occurred during the process of evolution of *G. hirsutum* and *G. barbadense*. For example, there was a duplication event on chromosome 1 in *G. hirsutum* and a loss event on chromosome 10 in *G. barbadense.* Overall, *SKP1-like* genes in cotton are relatively evolutionarily well conserved.

### 3.2. GhSKP1-like Genes Regulate Various Aspects of Stress Response

Previous research revealed that members of the *SKP1-like* family play a critical role in regulating the response to stress conditions [20]. In soybean, *GmSK1* (an SKP1 homolog)-overexpressing transgenic tobacco plants presented increased tolerance to high salinity and drought stress [21]. *GsSKP21* overexpression increased the tolerance of soybean plants to alkaline stress and reduced their sensitivity to ABA [22]. Overexpression of *PSK1* in Arabidopsis exhibited enhanced tolerance to salinity, which was attributed to higher levels of proline and soluble sugars [23]. Compared with control plants, transgenic rice plants overexpressing OmSKP1 presented a more disease-sensitive phenotype [24]. Transcription analysis of the upstream 2000-bp sequence of the *GhSKP1-like* genes revealed the presence of various cis-acting elements associated with light, growth development, phytohormones, and stress, suggesting that the *GhSKP1-like* genes may be functionally diverse. Gene expression analysis revealed that the *GhSKP1-like* genes presented diverse patterns of expression across various tissues and in response to stress, and homologous genes presented similar expression characteristics. We identified seven genes that were not expressed across various tissues and after exposure to different stressors, suggesting that these genes may have either lost their function or been activated under specific environmental conditions. The expression of *GhSKP1-like_7A* and the homologous gene *GhSKP1-like_5D* significantly increased after 1 h of salt stress, and no significant differential expression was detected after other stresses, including PEG, cold, and VD injection. Furthermore, the plants presented salt sensitivity after gene silencing via VIGS, suggesting that these two homologous genes play important roles in the response to salt stress. Additionally, we discovered two genes that are not expressed in tissues and remain unexpressed after exposure to abiotic stresses. However, the expression of these genes was significantly elevated when the plants were subjected to VD stress. These results further illustrate that the *GhSKP1-like* genes exhibit functional diversity and specificity in response to stress.

### 3.3. SKP1-like Proteins Interact Specifically with F-Box Proteins

The SKP1 protein functions as a component of the SCF complex and interacts with F-box proteins to recognize substrates, thereby exercising a protein degradation function. In *G. hirsutum*, 592 F-box protein-encoding genes were identified [25]. However, 22 *GhSKP1-like* genes were identified in the present study. This finding implies that GhSKP1-like proteins need to recognize and bind to multiple structurally different F-box proteins. In wheat, Hong et al. reported different forms of interaction in six *TaSKP* with 3 F-boxes [15]. In *Physcomitrella patens*, 10 FBX proteins were selected for yeast two-hybrid with PpSKP1, and the results showed that PpSKP1 can interact with 4 of the FBX proteins [26]. In *A. thaliana*, 24 F-box proteins with different structures were constricted and their interaction with ASK (SKP1-like gene) was verified. ASK1, ASK2, ASK11, and ASK12 can interact with F-box proteins containing PAS/PAC, LRR, and unknown structures, but not interact with kelch-containing F-box proteins. However, only ASKl3 interacts with the F-box genes containing the kelch structure, but not with other F-box genes [27,28,29]. Interestingly, ASKl3 not only could bind to F-box proteins but also interact with non-SCF complex proteins [30]. These results suggested the specificity of SKP1-like protein binding to F-box protein and the complexity of SKP1-like protein function. In our study, Y2H experiments revealed that *GhSKP1-like_4D* and *GhSKP1-like_6A* can interact with *GhFB6* and *GhFB15*, whereas *GhSKP1-like_6D* can interact with only *GhFB6*. On the basis of the current findings, it is difficult to determine which F-box binds to *GhSKP1-like* to form the SCF complex to exercise its function, and further biochemical assays are needed for confirmation.

## 4. Materials and Methods

### 4.1. Plant Material and Growth Conditions

The *G. hirsutum* cultivar “J172” was selected for expression analysis. Cotton seeds were sown in nutrient soil or nutrient solution. Seedlings were grown at 25 °C under 16 h light and 8 h dark photoperiods. When the cotton seedlings reached the “three leaves and one heart” stage, a Verticillium dahliae spore suspension was injected into the roots of the cotton in nutrient soil. Cotton seedlings in nutrient solution were treated with 200 mM NaCl. The leaves of the cotton seedlings were taken at 0, 1, 3, 6, 12, and 24 h. For each treatment and time point, three biological replicates were prepared and stored at −80 °C.

### 4.2. Identification and Sequence Analysis of SKP1-like Proteins in Cotton

The genome data of different cotton species were downloaded from the Gossypium Resource and Network Database (GRAND [31], https://grand.cricaas.com.cn/ (accessed on 31 December 2019)). Then, the hidden Markov model file of the *SKP1-like* gene family (PF01513) was downloaded from PFAM [32] (http://pfam.xfam.org/, accessed on 1 May 2022) website and retrieved via HMMER 3.3.2 [33]. Finally, sequences with intact SKP1 domains were screened and named sequentially on the basis of their position on the chromosome. The molecular masses and isoelectric points were calculated via the Compute pI/Mw tool of ExPaSy (https://web.expasy.org/compute_pi/ (accessed on 15 March 2022)). The subcellular localization of *SKP1-like* genes was predicted via WoLF PSORT (http://www.genscript.com/psort/wolf_psort.html (accessed on 17 February 2022)).

### 4.3. Phylogenetic Tree, Gene Structure, Conserved Motif, and Promoters cis-Acting Elements and TFs Analysis of the SKP1-like Gene Family

The phylogenetic tree contained full-length amino acid sequences of SKP1-like from four cotton species. All of the full-length amino acid sequences were aligned with ClustalX [34,35] via default parameters. MEGA11 software (version 11.0.13) [36] was used to generate the neighbor-joining phylogenetic tree with the following parameters: 1000 bootstrap tests, Poisson models, and pairwise deletions. The classification of cotton SKP1-like proteins was based on the topology and bootstrap values of the phylogenetic tree. The motif patterns were generated via TBtools version 2.083 software [37] (https://github.com/CJ-Chen/TBtools (accessed on 2 March 2022)). The upstream sequence (2.0 kb) of the GhSKP1-like sequence was retrieved via TBtools software (version 2.102) and then submitted to PlantCARE for cis-element analysis (http:// bioinformatics.psb/webtools/ plantcare/html/ (accessed on 17 December 2021)) [38].

### 4.4. Chromosome Location and Collinearity Analysis

The localization of chromosomes was accomplished using TBtools Gene Location Visualize from GTF/GFF. Collinearity analysis of the repetitive gene pairs of 4 cotton species (*G. barbadense*, *G. hirsutum*, *G. arboreum*, and *G. raimondii*) was performed via TBtools One step Mcscanx [39]. The chromosome length file and the genome alignment file were used for visualization of the collinearity results.

### 4.5. Gene Expression Analysis Based on RNA Sequencing

The tissue expression data for *GhSKP1-like* genes (FPKM) were obtained from the GRAND and Genome Sequence Archive (GSA, https://ngdc.cncb.ac.cn/gsa/ (accessed on 13 December 2024)), and the data were analyzed and visualized via Paiseno’s cloud platform (https://www.genescloud.cn/chart/CorHeatmap (accessed on 24 September 2024)). The data were standardized against various genes and then hierarchically clustered according to the phylogenetic tree. FPKM (Fragments Per Kilobase of transcript per Million mapped reads) is an expression-level normalization method. The FPKM normalizes read count based on gene length and the total number of mapped reads, which can reflect the expression level of a gene in the sample; the larger the FPKM value, the higher the gene expression level. To standardize the relative expression levels of each homolog among the gene pairs, we adjusted the absolute FPKM for each gene within those pairs in the following manner: expression in tissue = log2 (FPKM + 1); stress expression = log2 (fold change).

### 4.6. RNA Extraction and qRT–PCR Analysis

Relative mRNA expression was determined via RT–qPCR analysis. Total RNA was extracted via a plant RNA kit (R6827-01) (Omega Biotek, Guangzhou, China) and then subjected to reverse transcription via MonScriptTM RTIll Super Mix with dsDNase (two-step) (Monad, Suzhou, China). Quantitative real-time PCR was carried out using ChemoHS qPCR Mix (Monad, Suzhou, China). RT–qPCR was performed via a real-time PCR detection system (Bio-Rad, Hercules, CA, USA). The cycling conditions were as follows: 3 min hot start at 95 °C, followed by 40 cycles of denaturation at 95 °C for 30 s and annealing and extension at 58 °C for 30 s. Histone was used as the reference gene. Relative gene expression levels were measured via the comparative Ct(2^−ΔΔCt^) method. The real-time qPCR primer sequences are provided in Additional Table S1. Each sample was run in triplicate.

### 4.7. Virus-Induced Gene Silencing Assays

For VIGS, 10-day-old cotton seedlings were infiltrated with TRV-based VIGS vectors (pTRV2) containing target genes or with the control vector TRV2:00, as described previously. The *Agrobacterium tumefaciens* strain GV3101 (containing the *GhSKP1-like* gene and TRV1) vector was used for the VIGS experiments. *Agrobacterium tumefaciens* was grown at 28 °C on Luria–Bertani (LB) broth supplemented with appropriate antibiotics. Agrobacterium inoculation buffer (10 M MgCl_2_, 10 M MES pH 5.6, 150 μM acetosyringone) was adjusted to a final OD600 of 1.0 (for both the TRV1 and TRV2-*GhSKP1-like* genes), and the mixture was shaken for 4–6 h at room temperature before infiltration. For leaf infiltration, each Agrobacterium strain containing TRV1 and TRV2 vectors was mixed at a 1:1 ratio and infiltrated into the cotyledons (fully expanded) with a needleless syringe. The infiltrated plants were monitored for approximately 10 d. This experiment had 20 replicates and one plant per replicate. The experiment was repeated three times with similar results.

### 4.8. Binary Yeast Two-Hybrid Analysis

Yeast two-hybrid analysis was used to examine the interaction of SKP1-like/F-box proteins. The CDSs of *GhFB15* and *GhFB6* were subsequently cloned and inserted into pGBKT7, and the *GhSKP1-like* genes were subsequently cloned and inserted into pGADT7 (ClonExpress^®^ II One Step Cloning Kit) (Vazyme, Nanjing, China). In accordance with the instructions of the yeast transformation kit, the plasmid was transformed via the lithium acid method (Yeast Transformation Kit) (Coolaber, Beijing, China). Cotransformed yeast cells were selected by growing on media lacking leucine and tryptophan. The interaction between F-box proteins and GhSKP1-like proteins was monitored by growth on SD but without leucine, tryptophan, adenine, or histidine.

## 5. Conclusions

In this study, we performed a genome-wide identification of the *SKP1-like* gene family and revealed that 16 *GhSKP1-like* genes were expressed in response to biotic or abiotic stresses. The VIGS results showed that the silencing of *GhSKP1-like_7A* resulted in the inhibition of plant growth under salt stress, suggesting that *GhSKP1-like_7A* was involved in the response to salt stress. In addition, yeast two-hybrid results revealed that GhSKP1-like proteins have different abilities to interact with F-box proteins. The findings offer important insights for clarifying the evolutionary connections of the SKP1-like gene family and supporting additional research on the roles of SKP1-like genes in cotton.

## Data Availability

The original contributions presented in this study are included in the article and Supplementary Materials. Further inquiries can be directed to the corresponding authors.

## Supplementary Materials

The following supporting information can be downloaded at: https://www.mdpi.com/article/10.3390/ijms26010418/s1.
